# Does *Legionella* 5S rRNA PCR melting temperature analysis discriminate high-risk species and increased disease severity or does it simply reflect test selection bias?

**DOI:** 10.1128/jcm.01069-26

**Published:** 2026-10-27

**Authors:** Paul H. Edelstein

**Affiliations:** 1 Department of Pathology and Laboratory Medicine, Perelman School of Medicine University of Pennsylvania 6572, Philadelphia, Pennsylvania, United States; 2 Department of Medicine, University of Cambridge, Cambridge, United Kingdom; National Institute of Allergy and Infectious Diseases Division of Intramural Research, Bethesda, Maryland, USA

**Keywords:** test selection bias, Legionnaires' disease, *Legionella*

## LETTER

Pulsipher and colleagues state that patients with a positive respiratory sample 5S rRNA PCR assay for *Legionella* spp., with a melting point result >66°C, are at higher risk of severe Legionnaires’ disease (LD) than are those with positive tests but with a lower melting point result (1). They present good evidence that *L. pneumophila* and *L. Longbeachae* are responsible for positive PCR assays with higher melting points. Both *L. pneumophila* and *L. Longbeachae* cause LD in both immunocompromised and non-immunocompromised patients, whereas the other *Legionella* spp. causes disease almost exclusively in immunocompromised patients (2). Since immunocompromised patients are reported to have worse LD clinical outcomes than those without immunocompromise (2, 3), their conclusion that *L. pneumophila* and *L. longbeachae* infections have greater disease severity seems at odds with what is understood to be the case. One trivial explanation of this is that non-immunocompromised patients with community-acquired pneumonia (CAP) are unlikely to be tested for LD unless they are severely ill. The CAPNETZ prospective CAP study showed that about half of those with LD were outpatients who had relatively mild disease (4). Similar findings were reported by Foy et al., who found that outpatient LD was common (5). In contrast, it may be more likely that immunocompromised patients with CAP of all severities are tested for LD. It is likely that the Pulsipher et al. study detected only a very small fraction of cases of either *L. longbeachae* or *L. pneumophila* cases, almost all of whom had testing done only as hospitalized inpatients, inflating the apparent severity of disease caused by these two *Legionella* spp. Knowing LD diagnostic testing practices in their patient cohort could inform their conclusions to help determine whether this apparent association of severity with *L. pneumophila* and *L. longbeachae* LD is simply the result of testing bias or a significant indicator of species-specific virulence. Can the authors provide data on when LD testing was done in relation to severity of illness on presentation and on the frequency of LD testing in patients with CAP who were either immunocompromised or immunocompetent? Regardless of the presence of testing bias, the conclusion that *L. longbeachae* LD is associated with increased severity must be considered to have weak supporting evidence because only two patients in their cohort were known to have had LD caused by this *Legionella* spp.
